# Supplementary material for: L2C2: Last-level compressed-contents non-volatile cache and a procedure to forecast performance and lifetime
Source: PLoS One. 2023 Feb 7;18(2):e0278346. doi: 10.1371/journal.pone.0278346 (PMC9904472; doi:10.1371/journal.pone.0278346)
Supplement: S1 Appendix — (PDF) [file pone.0278346.s001.pdf]

S1 Appendix. Time scaling of forecasted indexes when considering bitcells with more endurance.

Let

$$N(w_b;\ \mu,\ \sigma) = \frac{1}{\sigma\sqrt{2\pi}} e^{\frac{-(w_b-\mu)^2}{2\sigma^2}} \tag{1}$$

be the normal probability distribution function that estimates the number of writes w_b causing failure in a *baseline* bitcell. Assuming a constant write rate WR (writes/s) on the baseline bitcell, the probability distribution function of the failure time t_b can be obtained from Eq. 1 by the linear transformation $t_b = \frac{w_b}{\mathrm{WR}}$:

$$N(t_b;\ \frac{\mu}{\mathrm{WR}},\ \frac{\sigma}{\mathrm{WR}}) \tag{2}$$

We can characterize an *improved* bitcell, with k times higher endurance, by applying to Eq. 1 and 2 the linear transformation $w_i = w_b \cdot k$. Thus, the probability distribution functions of the number of writes and failure time for the improved bitcell are, respectively:

$$N(w_i;\ \mu \cdot k,\ \ \sigma \cdot k) \quad \text{and} \quad N(t_i;\ \frac{\mu \cdot k}{\mathrm{WR}},\ \frac{\sigma \cdot k}{\mathrm{WR}}) \tag{3}$$

On the other hand, the probability of failure of the baseline bitcell, P_b, at a time $t_b \leq t$ is:

$$P_b(t_b \leq t) = \int_0^t N(t_b;\ \frac{\mu}{\mathrm{WR}},\ \frac{\sigma}{\mathrm{WR}}) dt_b \tag{4}$$

To know the probability of failure of the improved bitcell, P_i, at a time $t_i \leq t$ from P_b, it is necessary to apply another linear transformation: $t_b = \frac{t_i}{k}$. Thus:

$$P_i(t_i \leq t) = P_b(t_b \leq \mathrm{lin\_trans}_{i \to b}(t)) = P_b(t_b \leq \frac{t}{k})) \tag{5}$$

Rewriting the two probabilities as a function of t, we have:

$$P_i(t) = P_b(\frac{t}{k}) \tag{6}$$

To conclude, let us consider a cache with c baseline bitcells, each with an endurance approximated by the probability distribution of Eq. 1 and subjected to a constant per-cell write rate WR (writes/s). Assuming bit granularity the decrease of its effective capacity with time, $\mathrm{Ceff}_b(t)$ is:

$$\mathrm{Ceff}_b(t) = C \cdot (1 - P_b(t)) \tag{7}$$

And for a cache of the same size made with improved bitcells:

$$\mathrm{Ceff}_i(t) = C \cdot (1 - P_b(\frac{t}{k})) \tag{8}$$

In this case, with byte granularity and different write rates in each frame, it can be reasoned in the same way. That is, any forecasted index with enhanced cells at time t matches the same index forecasted with base cells but at time $\frac{t}{k}$.
